# Supplementary material for: V-Cornea: A computational model of corneal epithelium homeostasis, injury, and recovery
Source: bioRxiv. 2025 Aug 14:2025.08.11.669602. Preprint. [Version 1] doi: 10.1101/2025.08.11.669602 (PMC12363777; doi:10.1101/2025.08.11.669602)
Supplement: 1 [file NIHPP2025.08.11.669602v1-supplement-1.pdf]

bioRxiv preprint doi: https://doi.org/10.1101/2025.08.11.669602; this version posted August 14, 2025. The copyright holder for this preprint (which was not certified by peer review) is the author/funder, who has granted bioRxiv a license to display the preprint in perpetuity. It is made available under aCC-BY 4.0 International license.

S1. Growth Dynamics Mathematical Formulation

S1.1 EGF-Dependent Growth (Hill Function)

For basal and stem cell types, the growth factor due to EGF concentration is defined as:

$$G_{nEGF,i} = \left(\frac{EGF_{n,i}{}^{4}}{k_{mEGF,i}{}^{4} + EGF_{ni}{}^{4}}\right)(1)$$

where EGF_{ni} is the average EGF concentration in cell i at time n. We define:

$$EGF_{ni} = \frac{\vartheta_{nEGF,i}}{V_{ni}}(2)$$

where

$$\vartheta_{nEGF,i} = \sum_{p\in i} F_{EGF}(p)(3)$$

i.e.,$\vartheta_{nEGF,i}$ is the total EGF amount inside cell i, obtained by summing the EGF field F_{EGF} over all lattice pixels p belonging to cell i. V_{ni} is the cell's volume (in number of pixels or voxels) at time n. The parameter $k_{mEGF,i}$ is the half-maximal EGF concentration for cell i.

S1.2 Density-Dependent Growth Inhibition (Hill Function)

Cells experience density-dependent growth inhibition described by another Hill-type function:

$$G_{ndensity,i} = \left(\frac{k_{mdensity,i}{}^{4}}{k_{mdensity,i}{}^{4} + P_{ni}{}^{4}}\right)(4)$$

where P_{ni} is the effective "pressure" inside cell i at time n, derived from its volume deviation (see below). The parameter $k_{mdensity,i}$ is the half-maximal pressure for growth inhibition.

S1.3 Effective Pressure Calculation

The volume energy term in the Hamiltonian is:

$$H_{volume,i} = \lambda_{v,i}\left(V_i - V_{target,i}\right)^2(5)$$

where $\boldsymbol{\lambda_{v,i}}$ is the volume constraint parameter for cell i; $\boldsymbol{V_i}$ is the actual (current) volume of the cell $\boldsymbol{i}$**,** and $\boldsymbol{V}_{target,\boldsymbol{i}}$ is the target volume of the cell $\boldsymbol{i}$.

The effective pressure inside cell i is then:

bioRxiv preprint doi: https://doi.org/10.1101/2025.08.11.669602; this version posted August 14, 2025. The copyright holder for this preprint (which was not certified by peer review) is the author/funder, who has granted bioRxiv a license to display the preprint in perpetuity. It is made available under aCC-BY 4.0 International license.

$$P_{ni} = \frac{\partial H_{volume,i}}{\partial V_i} = 2\lambda_{v,i}\left(c - V_{target,i}\right)(6)$$

this pressure term reflects how far the cell's volume is from its target.

S1.4 Total Growth Rate

Combining the EGF-dependent growth and density-dependent inhibition terms, each cell's net growth rate is:

$$G_{ntotal,i} = \delta_i \times G_{ndensity,i} \times G_{nEGF,i}(7)$$

where δ_i is the cell's intrinsic maximal doubling rate (e.g., δ_{basal} or δ_{stem}) given in hours. Finally, cell i grows by updating its target volume:

$$V_{targetn+1i} = V_{targetni} + G_{ntotal,i}(8)$$

S1.5 Parameter Fitting for Hill Function Coefficients

The half-maximal concentration parameters (k_{mEGF} and $k_{mdensity}$) are critical control points in our growth regulation model, directly influencing proliferation rates in response to both EGF signaling and contact inhibition. To determine appropriate values for these parameters, we employed a multi-objective optimization approach constrained by three key biological criteria:

1. **Epithelial Turnover and Recovery Timeline**: Values were calibrated to ensure that complete epithelium turnover time of 7-14 days, and the recovery of 3-5 days after injury, matched the empirically observed times in healthy corneal epithelium (7,84).
2. **Layer-Specific Cell Density**: Parameter values were adjusted to maintain physiologically relevant cell numbers, 5–7 layers of cells (100).
3. **Epithelial Thickness Maintenance**: We constrained our parameters to maintain stable epithelial thickness of 54 ± 5 μm over extended simulations, consistent with in vivo measurements (100).

We performed an iterative parameter sweep, systematically varying k_{mEGF} from 1.0 to 10.0 and $k_{mdensity}$) from 10.0 to 200.0 across multiple simulation runs. The optimal values ($k_{mEGF,\,stem} = 3.5$, $k_{mEGF,\,basal} = 7.0$ and $k_{mdensity}$ = 125.0 for both) produced the closest match to all three biological constraints simultaneously.

bioRxiv preprint doi: https://doi.org/10.1101/2025.08.11.669602; this version posted August 14, 2025. The copyright holder for this preprint (which was not certified by peer review) is the author/funder, who has granted bioRxiv a license to display the preprint in perpetuity. It is made available under aCC-BY 4.0 International license.

S1.6 Mitosis Rule

Mitosis in our model is governed by a volume-threshold mechanism that initiates cell division once a cell accumulates sufficient biomass.

Formally, for any proliferative cell i (stem or basal), division occurs when:

$$P_{divide} = \begin{cases} 1, & V_i > \omega_{v,i} \\ 0, & otherwise \end{cases} \quad (9)$$

where V_i represents the current volume of cell, and$\omega_{v,i}$ is the cell type-specific volume threshold for division. We set $\omega_{v,i} = 2V_{0_i}$ where V_{0_i} is the initial cell volume (25 pixels or approximately 100 μm²).

When a cell reaches this threshold, we implement division through the following algorithm:

1. Identify the division plane orientation:

For limbal epithelial stem cells (LESCs), we orient the division plane to direct daughter cells centripetally, following observed patterns that maintain stem cell populations while producing committed progenitors (64).

For basal cells, we select a random division plane to promote even tissue distribution (48).

2. Create two daughter cells with equal volumes:

$$V_{d1} = V_{d2} = \frac{V_p}{2} (10)$$

where V_{d1} and V_{d2} are the volumes of the daughter cells, and V_p is the parent cell's volume at division.

3. Reset target volumes for daughter cells to their initial type-specific values:

$$V_{targetd1} = V_{targetd2} = V_{0target,i} (11)$$

4. Preserve cell type-specific properties:

Each LESC division produces one stem cell and one basal cell, maintaining the stem cell pool while generating committed progenitors (83).

Basal cell divisions produce two identical basal cells, both capable of further proliferation.

bioRxiv preprint doi: https://doi.org/10.1101/2025.08.11.669602; this version posted August 14, 2025. The copyright holder for this preprint (which was not certified by peer review) is the author/funder, who has granted bioRxiv a license to display the preprint in perpetuity. It is made available under aCC-BY 4.0 International license.

S2. Differentiation Rules Mathematical Formulation

S2.1 Stem to Basal Differentiation

A limbal epithelial stem cells (LESCs) differentiates into basal cells it loses contact with limbal Bowman's layer / Epithelial Basement Membrane (EpBM):

$$P_{(STEM \to BASAL)} = \begin{cases} 1, & if\ \neg(LIMB \in N) \\ 0, & otherwise \end{cases} \quad (12)$$

here N is the set of neighboring cell types, $\neg LIMB \in N$ means the LIMB type is not in the neighbor set.

S2.2 Basal to Wing Differentiation

Basal cells differentiate to wing cells based on their contact area with Bowman's layer/Epithelial Basement Membrane (EpBM):

$$P_{(BASAL \to WING)} = \begin{cases} 1, & if\, \mathrm{A}_{\mathrm{EpBM}} \leq \omega_{\mathrm{contact,basal}} \\ 0, & otherwise \end{cases} (13)$$

where A_{EpBM} is the number of pixels in contact with the basement membrane, and $\omega_{contact,basal}$ $= 5$ pixels are the minimal contact area required to maintain basal phenotype.

S2.3 Wing to Superficial Differentiation:

A wing cell differentiates into a superficial cell once it contacts the surface (tear film) and loses contact with deeper layers. The transition is:

$$P_{(\mathrm{WING} \to \mathrm{SUPER})} = \begin{cases} 1, & if\,(TEAR \in N) \wedge (WING \in N) \wedge \neg(BASAL \in N) \wedge \neg(MEMB \in N) \wedge \neg(STEM \in N) \\ 0, & otherwise \end{cases} (14)$$

$$P_{(\mathrm{WING} \to \mathrm{SUPER})} = \begin{cases} 1, & \text{if } \{\text{TEAR,WING}\} \subseteq \text{N and } \{\text{BASAL, MEMB,STEM}\} \cap \text{ N} \\ 0, & otherwise \end{cases} (14)$$

All these transitions are deterministic (P=1 or P=0) and occur immediately once the condition is met.

bioRxiv preprint doi: https://doi.org/10.1101/2025.08.11.669602; this version posted August 14, 2025. The copyright holder for this preprint (which was not certified by peer review) is the author/funder, who has granted bioRxiv a license to display the preprint in perpetuity. It is made available under aCC-BY 4.0 International license.

S3. Movement Implementation in the Cellular Potts Model

S3.1 Hamiltonian Definition

Cell movement is governed by the minimization of the system's Hamiltonian:

$$\Delta H = \Delta H_{contact} + \Delta H_{links} + \Delta H_{volume} + \Delta H_{surface} + \Delta H_{chemotaxis} \quad (15)$$

S3.2 Contact and Links Energy Terms

The contact energy is calculated as:

$$H_{contact} = \sum_{(p,q)} J\left(\tau(\sigma(p)), \tau(\sigma(q))\right) \quad (16)$$

where $J(\tau_1, \tau_2)$ is the contact energy between cell types τ_1 and τ_2 and $\sigma(p)$ is the cell occupying lattice site p. $\tau(\sigma)$ gives the cell type of cell σ.

Each link is modeled as a Hooke-type (spring) interaction between the centers of mass of two cells (or between one cell and a "wall" cell). These springs can be created, updated (e.g., to maintain a certain tension), or removed depending on local conditions in the simulation.

$$H_{links} = \lambda_{di,j}\left(d_{i,j} - d_{targeti,j}\right)^2 \quad (17)$$

Where $\lambda_{di,j}$ is the link`s spring constant, $d_{i,j}$ is the current distance (in voxels) between the cell centers, $d_{targeti,j}$ is the links equilibrium length.

From this energy, the tension (force) on each cell due to the link is:

$$\gamma_{i,j} = \frac{\partial H_{links}}{\partial d_{i,j}} = 2\lambda_{i,j}\left(d_{i,j} - d_{target_{i,j}}\right) \quad (18)$$

We enforce a constant tension on the superficial cells layer by automatically adjusting either $\lambda_{i,j}$ or $d_{target_{i,j}}$.

$$\gamma_{i,j} = 2\lambda_{i,j}\left(d_{i,j} - d_{target_{i,j}}\right) = \gamma_{target} \quad (19)$$

Case of Adjusting $\boldsymbol{\lambda_{i,j}}$

$$\lambda_{i,j} = \frac{\frac{\gamma_{target}}{2}}{d_{i,j} - d_{target_{i,j}}} \quad (20)$$

Case of Adjusting $\boldsymbol{d_{target_{i,j}}}$

bioRxiv preprint doi: https://doi.org/10.1101/2025.08.11.669602; this version posted August 14, 2025. The copyright holder for this preprint (which was not certified by peer review) is the author/funder, who has granted bioRxiv a license to display the preprint in perpetuity. It is made available under aCC-BY 4.0 International license.

$$d_{target_{i,j}} = \frac{\gamma_{target}}{2\lambda_{i,j}} \quad (21)$$

Depending on the user's "AutoAdjustLinks" and "Lambda_link_adjustment" settings, the code picks one of these two strategies to keep $\gamma_{i,j}$ at the desired tension.

S3.3 Volume and Surface Constraints

Volume and surface energy terms are:

$$H_{volume} = \lambda_v \sum_i \left(V_i - V_{targeti}\right)^2 \quad (22)$$

$$H_{surface} = \lambda_s \sum_i \left(S_i - S_{targeti}\right)^2 \quad (23)$$

Where λ_v, λ_s are volume/surface coefficients; V_i, S_i are the current volume/surface area. V_{target_i}, S_{target_i} are the respective targets.

S3.4 Chemotaxis Term

The chemotactic energy contribution is:

$$\Delta H_{chemotaxis} = -\lambda_c\left(c(x') - c(x)\right) \quad (24)$$

Where λ_c is the chemotactic coefficient, and $c(x)$ is the chemical concentration at position x. A proposed copy from x to x' changes the Hamiltonian according to ΔH.

S3.5 Movement Algorithm

1. Randomly choose a lattice pixel x and one of its neighbors x'.
2. Compute ΔH for copying $\sigma(x) \rightarrow \sigma(x')$.
3. Accept with probability

$$P_{accept} = \begin{cases} 1, & \Delta H \le 0 \\ exp\left(-\frac{\Delta H}{T}\right), & \Delta H > 0 \end{cases} \quad (25)$$

where T is the simulation temperature parameter.

bioRxiv preprint doi: https://doi.org/10.1101/2025.08.11.669602; this version posted August 14, 2025. The copyright holder for this preprint (which was not certified by peer review) is the author/funder, who has granted bioRxiv a license to display the preprint in perpetuity. It is made available under aCC-BY 4.0 International license.

S4. Cell Death and Sloughing Mathematical Formulation

S4.1 Natural Cell Death (Sloughing)

For superficial cells in contact with tear film, the probability of sloughing is:

$$P_{slought} = U(0,1) < \left(\frac{1}{3} * DaytoMCS\right) (26)$$

where $U(0,1)$ is a uniform random sample in [0,1], and $DaytoMCS$ is the factor converting between days and Monte Carlo Steps. The multiplier 1/3 is calibrated so that the average layer transit time is about 1.75 days out of a total 7–14 day turnover cycle.

S4.2 Volume-Based Death Condition

For superficial cells:

$$P_{death} = \begin{cases} 1, & if\ V_{ni} < \omega_{volume,super}\ or\ N_{super} = 0 \\ 0, & otherwise \end{cases} (27)$$

where $V_{ncell,i}$ is current cell volume, $\omega_{volume,super} =\ 15$ (minimum viable cell size), N_{super} is the number of neighboring superficial cells.

S4.3 Volume Reduction During Death

Dying cells shrink to zero volume:

$$\frac{\partial V_i}{\partial t} = -\lambda_{v_i}\left(V_i - V_{targeti}\right)(28)$$

where for a dying cell, $V_{target_i} = 0$ and λ_{v_i} is increased to 1000 for rapid shrinkage.

S4.4 Injury Implementation

A. Ablation Injuries

Ablation injuries are simulated by the instantaneous removal of cells within a defined circular region. Any part of the cell included in the circular area will be selected to be part of the cells set to be removed, defined as:

$$S_{ablate} = \{(x,y) \mid \sqrt{(x - x_{center})^2 + (y - y_{center})^2} \leq (29)$$

All cells intersecting S_{ablate} are converted to "tear" type:

$$\forall C \in S_{ablate}, \text{then } C_i \rightarrow C_{tear} (30)$$

simulating the removal of epithelium and its replacement by tear fluid in the wound, as observed in corneal wound healing (Wilson et al., 1999).

bioRxiv preprint doi: https://doi.org/10.1101/2025.08.11.669602; this version posted August 14, 2025. The copyright holder for this preprint (which was not certified by peer review) is the author/funder, who has granted bioRxiv a license to display the preprint in perpetuity. It is made available under aCC-BY 4.0 International license.

B. Chemical Injuries

Chemical injuries are modeled through a reaction-diffusion equation:

$$\frac{\partial c_{chem}}{\partial t} = \vec{\nabla} \cdot \left(D_{chem} \vec{\nabla} c_{chem}\right) - k_{d_{chem}} * c_{chem} (31)$$

where $c_{chem}(x,y,t)$ is chemical concentration, D_{chem} is the diffusion coefficient that is dependent on localization, $k_{d_{chem}}$ is degradation rate.

With two initial-condition types:

Gaussian Pulse (droplet or localized exposure):

$$c_{chem}(x,y) = c_{0_{chem}} * exp\left(-\left((x - x_{center_{chem}})^2 + (y - y_{center_{chem}})^2\right) / 2\sigma^2\right) (32)$$

where $c_{0_{chem}}$ is initial peak concentration, σ is distribution width parameter, ($x_{center_{chem}}$, $y_{center_{chem}}$) is exposure center point.

Uniform Distribution (coating or widespread exposure):

$$c_{chem}(x,y) = \frac{c_{0_{chem}}}{L_x} \; for\ all\ x \in [0,L_x] \times y_{center_{chem}} (33)$$

where L_x are the whole lattice dimension in x, $y_{center_{chem}}$ is the given height, and $c_{0_{chem}}$ is initial concentration.

S4.5 Cell Death Conditions

Cells die if their mean chemical concentration exceeds a threshold. Let

$$\vartheta_{nchem,i} = \sum_{p \in i} F_{chem}(p) (34)$$

$$Chem_{ni} = \frac{\vartheta_{nchem,i}}{V_{ni}} (35)$$

$F_{chem}(p)$ is the chemical field value at pixel p within the cell, $\vartheta_{nchem,i}$ is the total amount of chemical observed by the cell i at time n, V_{ni} is the volume of the cell i, lastly $Chem_{ni}$ is the average concentration of chemical at cell i at time n. Which define the set of doomed cells in:

$$S_{chem} = \{i \mid Chem_{ni} > \omega_{chem} (36)$$

All cells in S_{chem}, undergo rapid volume collapse:

$$\forall C \in S_{chem} : \begin{matrix} \lambda_v = 1000 \\ V_{target} = 0 \end{matrix} (37)$$

bioRxiv preprint doi: https://doi.org/10.1101/2025.08.11.669602; this version posted August 14, 2025. The copyright holder for this preprint (which was not certified by peer review) is the author/funder, who has granted bioRxiv a license to display the preprint in perpetuity. It is made available under aCC-BY 4.0 International license.

S5. EGF Dynamics Mathematical Formulation

S5.1 Spatiotemporal Evolution

The EGF concentration field $c_{EGF}(x,y,t)$ evolves according to the reaction-diffusion equation:

$$\frac{\partial c_{EGF}}{\partial t} = D_{EGF}(x,y)\nabla^2 c_{EGF} - k_{d_{EGF}} * c_{EGF} + \Omega_{EGF}(x,y,t) \quad (38)$$

where $D_{EGF}(x,y)$ is the cell-type dependent diffusion coefficient, $k_{d_{EGF}} = 0.5\ MCS^{-1}$ ($EGF_{GlobalDecay}$) is the global decay rate, $\Omega_{EGF}(x,y,t)$ represents the net source/sink terms.

S5.2 Cell-Type Dependent Transport

EGF diffusion coefficient varies by cell type:

$$D_{EGF}(x,y) = \begin{cases} 20.0, & in\ superficial\ cells\left(D_{\mathrm{EGF,super}}\right) \\ 20.0, & in\ membrane\ EpBM\left(D_{\mathrm{EGF,memb}}\right) \\ 0, & in\ limbal\ EpBM\left(D_{\mathrm{EGF,limb}}\right) \\ \mathrm{D}_{\mathrm{global}_{\mathrm{EGF}}} & otherwise \end{cases} \quad (39)$$

$\mathrm{D}_{\mathrm{global}_{\mathrm{EGF}}}$ global diffusion constant for EGF was set at 186 voxels²/MCS based on experimental measurements adapted to our simulation parameters. We initially calculated the diffusion coefficient from rat brain tissue studies (101), yielding 466.2 voxels²/MCS after converting to our spatial (1 voxel = 2 μm) and temporal (1 hour = 10 MCS) scales. However, this value would cause unrealistically rapid equilibration across our simulation domain (200 × 90 voxels).

We therefore considered alternative data from GelMA hydrogel experiments (87), which reported a more restricted EGF diffusion coefficient (2.5×10^{-8} cm²/s), equivalent to 225 voxels²/MCS in our units. Our selected value of 186 voxels²/MCS closely approximates this experimentally determined rate for EGF in dense extracellular environments while remaining computationally feasible. This parameter allows for biologically relevant gradient formation, providing an appropriate balance between physiological accuracy and computational efficiency.

S5.3 Source and Sink Terms

$$\Omega_{EGF}(x,y,t) = \varphi_{EGF,Tear} - k_{d_{EGF}} - \sum_i \mu_{EGF,i} * c_{EGF}(x,y) \quad (40)$$

where $\varphi_{EGF,tear} = 1.0$ is the constant secretion rate by tear cells. And $\mu_{EGF,i}$ are cell-type specific uptake rates $\mu_{EGF,basal} = 0.0$ for basal cells, $\mu_{EGF,stem} = 0.0$ for stem cells, $\mu_{EGF,super} = 0.0$ for superficial cells, $\mu_{EGF,wing} = 0.0$ for wing cells, since we are using a increase global decay as a surrogate to these individual cells uptakes for simplicity.

bioRxiv preprint doi: https://doi.org/10.1101/2025.08.11.669602; this version posted August 14, 2025. The copyright holder for this preprint (which was not certified by peer review) is the author/funder, who has granted bioRxiv a license to display the preprint in perpetuity. It is made available under aCC-BY 4.0 International license.

The global decay constant for EGF $k_{d_{EGF}}$ was set to 0.5 per MCS to account for multiple biological processes affecting EGF availability. Using published data on EGF circulating half-life ranging from 42 to 114 minutes (88), we calculated corresponding decay constants between 0.099 and 0.036 per MCS in our simulation units (1 hour = 10 MCS).

Our implemented decay value (0.5) is intentionally higher than these physiological baseline values to implicitly incorporate additional processes not explicitly modeled, including cellular uptake, receptor-mediated endocytosis, proteolytic degradation, and sequestration by extracellular matrix components. This higher decay rate also ensures appropriate diffusion length scales within our simulation domain.

S5.4 Boundary Conditions

Physical Domain Structure

The simulation domain is bounded by non-diffusive wall cells at

$$x = 0 \ and \ x = L_x (wall\ cells) (41)$$

where wall cells have $D_{EGF,wall} = 0$ (no diffusion). These cells create effective no-flux boundaries by blocking EGF transport.

Mathematical Boundary Conditions

Horizontal Boundaries

Effective no-flux condition due to combination of:

1. Zero-derivative boundary condition at domain edges:

$$\frac{\partial c}{\partial x} = 0 \ \ at \ x = 0, L_x (42)$$

2. Impermeable wall cells creating physical barriers:

$$D = 0 \ \ in\ wall\ cells\ at\ x = 0, L_x (43)$$

Vertical Boundaries

Fixed concentration at top and bottom:

$$c = 0 \ at \ y = 0, L_y (44)$$

This configuration creates a physically confined system where:

EGF cannot escape through the lateral boundaries (wall cells + no-flux conditions)

Top and bottom boundaries maintain zero concentration

bioRxiv preprint doi: https://doi.org/10.1101/2025.08.11.669602; this version posted August 14, 2025. The copyright holder for this preprint (which was not certified by peer review) is the author/funder, who has granted bioRxiv a license to display the preprint in perpetuity. It is made available under aCC-BY 4.0 International license.

Effective transport is confined to the region between wall cells

S5.5 Spatial and Temporal Scales

Spatial:

Lattice spacing: $\Delta x = \Delta y = 1\, voxel = 2\, \mu m$

Domain size: $L_x = 200\, voxels, L_y = 90\, voxels$

Temporal:

1 Monte Carlo Step (MCS) = 6 minutes

bioRxiv preprint doi: https://doi.org/10.1101/2025.08.11.669602; this version posted August 14, 2025. The copyright holder for this preprint (which was not certified by peer review) is the author/funder, who has granted bioRxiv a license to display the preprint in perpetuity. It is made available under aCC-BY 4.0 International license.

Table S1. Stem cells behavior signal relationship

Agent Type	Behavior	Form	$\frac{Min}{Max}$	Signal(s)	Effect(s)	Params
**Stem**	Growth (Eq. 8)	Multiplicative Hill (Eq. 7)	$\frac{0}{\delta_{stem}}$	EGF (Eq. 1)	Increase	Half max: $k_{mEGF,stem}$<br>Hill power: 4
				Pressure (Eq. 4)	Decrease	Half max: $k_{mdensity,stem}$<br>Hill power: 4
	Differentiation to Basal (Eq. 12)	Boolean Conditional	0/1	Contact with Limbal BM	Disallow	$\omega_{contact,stem} = 1$ voxel
	Mitosis	Boolean Conditional	0/1	Cell Volume	Allow	$\omega_{v,stem} = 2V_{0target,\ stem}$
	Movement (Boltzmann Acceptance Eq. 25)	Contact Energy (Eq. 16)	$\frac{5}{20}$	Cell Neighbor	Energy Contribution	Table S6 energies
		Volume (Eq. 22)	$\frac{-\infty}{+\infty}$	Cell Volume	Energy Contribution	$\lambda_{0_{v,stem}}$=2.0,<br>$V_{0target,\ stem}$=25.0
		Surface Area (Eq. 23)	$\frac{-\infty}{+\infty}$	Cell Surface	Energy Contribution	$\lambda_{0_{s,stem}}$=2.0,<br>$S_{0target,stem}$=18.0
		Chemotaxis (Eq. 24)	$\frac{-\infty}{+\infty}$	Concentration Gradient	Increase Energy Contribution	$\lambda_{0_{chemo_{Mbias,stem}}} = 100$
	Apoptosis	Boolean Conditional (Eq. 36)	0/1	Chemical Concentration	Allow	ω_{chem}

bioRxiv preprint doi: https://doi.org/10.1101/2025.08.11.669602; this version posted August 14, 2025. The copyright holder for this preprint (which was not certified by peer review) is the author/funder, who has granted bioRxiv a license to display the preprint in perpetuity. It is made available under aCC-BY 4.0 International license.

Table S2. Basal cells behavior signal relationship

Agent Type	Behavior	Form	$\frac{Min}{Max}$	Signal(s)	Effect(s)	Params
**Basal**	Growth (Eq. 8)	Multiplicative Hill (Eq. 7)	$\frac{0}{\delta_{basal}}$	EGF (Eq. 1)	Increase	Half max: $k_{mEGF,basal}$ Hill power: 4
				Pressure (Eq. 4)	Decrease	Half max: $k_{mdensity,basal}$ Hill power: 4
	Differentiation to Wing (Eq. 10)	Boolean Conditional	0/1	Contact with Periphery BM	Disallow	$\omega_{contact,basal} = 5$ voxels
	Mitosis	Boolean Conditional	0/1	Cell Volume	Allow	$\omega_{v,basal} = 2V_{0target,basal}$
	Movement (Boltzmann Acceptance Eq. 25)	Contact energy (Eq. 16)	$\frac{5}{20}$	Cell Neighbor	Energy Contribution	Table S6 energies
		Volume (Eq. 22)	$\frac{-\infty}{+\infty}$	Cell Volume	Energy Contribution	$\lambda_{0_{v,basal}} = 2.0$, $V_{0target,basal} = 25.0$
		Surface area (Eq. 23)	$\frac{-\infty}{+\infty}$	Cell Surface	Energy Contribution	$\lambda_{0_{s,basal}} = 5.0$, $S_{0target,basal} = 25.0$
		Chemotaxis (Eq. 24)	$\frac{-\infty}{+\infty}$	Concentration Gradient	Increase	$\lambda_{0_{chemo_{Mbias,basal}}} = 1000$
	Apoptosis	Boolean Conditional (Eq. 36)	0/1	Chemical Concentration	Allow	ω_{chem}

bioRxiv preprint doi: https://doi.org/10.1101/2025.08.11.669602; this version posted August 14, 2025. The copyright holder for this preprint (which was not certified by peer review) is the author/funder, who has granted bioRxiv a license to display the preprint in perpetuity. It is made available under aCC-BY 4.0 International license.

Table S3. Wing cells behavior signal relationship

Agent Type	Behavior	Form	Min / Max	Signal(s)	Effect(s)	Params
**Wing**	Differentiation to Superficial (Eq. 11)	Boolean Conditional	0/1	Exposure to Tear, loss contact Basal	Allow	$\omega_{contact,wing-tear} > 0$ AND $\omega_{contact,wing-basal} < 1$
	Movement (Boltzmann Acceptance Eq. 25)	Contact energy (Eq. 16)	$\frac{5}{15}$	Cell Neighbor	Energy Contribution	Table S6 energies
		Volume (Eq. 22)	$\frac{-\infty}{+\infty}$	Cell Volume	Energy Contribution	$\lambda_{0_{v,wing}} = 2.0$, $V_{0target,wing} = 25.0$
		Surface area (Eq. 23)	$\frac{-\infty}{+\infty}$	Cell Surface	Energy Contribution	$\lambda_{0_{s,wing}} = 5.0$, $S_{0target,wing} = 25.0$
		Chemotaxis (Eq. 24)	$\frac{-\infty}{+\infty}$	Protein Concentration Gradient	Increase	$\lambda_{0_{chemo_{EGF,wing}}} = 20$
	Apoptosis	Boolean Conditional (Eq. 36)	0/1	Chemical Concentration,	Allow	ω_{chem}

Table S4. Superficial cells behavior signal relationship

Agent Type	Behavior	Form	Min / Max	Signal(s)	Effect(s)	Params
**Superficial**	Movement (Boltzmann Acceptance Eq. 25)	Contact energy (Eq. 16)	$\frac{2}{15}$	Cell Neighbor	Energy Contribution	Table S6 energies
		Volume (Eq. 22)	$\frac{-\infty}{+\infty}$	Cell Volume	Energy Contribution	$\lambda_{0_{v,super}}$=2.0, $V_{0target,super}$=25.0
		Surface area (Eq. 23)	$\frac{-\infty}{+\infty}$	Cell Surface	Energy Contribution	$\lambda_{0_{s,super}}$=5.0, $S_{0target,super}$=25.0
		Links (Eq. 17)	$\frac{-\infty}{+\infty}$	Center of Mass Distance	Energy Contribution	$\lambda_{0_{link,super}} = 50$<br>$L_{0_{target_{link,super}}} = 3$<br>$L_{m_{link,super}} = 1000$<br>$L_{0_{targetlink,wall}} = 3$<br>$\lambda_{0_{link,wall}} = 50$<br>$L_{m_{link,wall}} = 1000$
	Apoptosis	Boolean Conditional (Eq. 36)	0/1	Chemical Concentration	Allow	ω_{chem}
		Sloughing (Eq. 23)	0/1	Probability Draw	Allow	$\left(\frac{1}{3} * DaytoMCS\right)$

bioRxiv preprint doi: https://doi.org/10.1101/2025.08.11.669602; this version posted August 14, 2025. The copyright holder for this preprint (which was not certified by peer review) is the author/funder, who has granted bioRxiv a license to display the preprint in perpetuity. It is made available under aCC-BY 4.0 International license.

Table S5. Tear and Bowman`s/EpBM behavior signal relationship

Agent Type	Behavior	Form	$\frac{Min}{Max}$	Signal(s)	Effect(s)	Params
**Tear**	Movement (Boltzmann Acceptance Eq. 25)	Contact energy (Eq. 16)	$\frac{0.1}{10}$	Cell Neighbor	Energy Contribution	Table S6 energies
		Volume (Eq. 22)	$\frac{-\infty}{+\infty}$	Cell Volume	Energy Contribution	$\lambda_{0_{v,tear}}$=1.0, $V_{0_{target,tear}}$=50.0
	Secrete EGF	Constant	1	–	–	$\varphi_{EGFtear}$
**Bowman`s/ EpBM**	Destructible	Boolean Conditional (Eq. 36)	0/1	Chemical Concentration	Allow	ω_{chem}

Table S6 - Contact energy values (arbitrary units)

Cell Type	Medium	STEM	LIMB	BASAL	WING	SUPER	MEMB	STROMA	WALL	TEAR
**Medium**	10.0	10.0	10.0	10.0	10.0	10.0	10.0	10.0	5.0	5.0
**STEM**		10.0	5.0	10.0	10.0	10.0	5.0	20.0	10.0	5.0
**LIMB**			10.0	10.0	10.0	10.0	10.0	10.0	10.0	5.0
**BASAL**				10.0	10.0	15.0	5.0	20.0	10.0	5.0
**WING**					5.0	6.0	15.0	10.0	10.0	5.0
**SUPER**						2.0	15.0	10.0	5.0	2.0
**MEMB**							10.0	10.0	10.0	2.0
**STROMA**								10.0	10.0	10.0
**WALL**									10.0	5.0
**TEAR**										0.1

bioRxiv preprint doi: https://doi.org/10.1101/2025.08.11.669602; this version posted August 14, 2025. The copyright holder for this preprint (which was not certified by peer review) is the author/funder, who has granted bioRxiv a license to display the preprint in perpetuity. It is made available under aCC-BY 4.0 International license.

Table S7 – Fields descriptions

Field	Definition	Role / Properties	Units	Processes
F_{EGF}	long-diffusing proliferative factor	-regulates cell growth	nM	-diffuses<br>-decays
F_{Mbias}	short-diffusing chemoattractant	-regulates cell movement	nM	-diffuses<br>-uptake by Basal and Stem
F_{chem}	long-diffusing chemical injury	-regulates cell viability	scaled concentration (amount per voxel)	-diffuses<br>-uptake (cleared) by Tear<br>-uptake (cleared) by cell death

bioRxiv preprint doi: https://doi.org/10.1101/2025.08.11.669602; this version posted August 14, 2025. The copyright holder for this preprint (which was not certified by peer review) is the author/funder, who has granted bioRxiv a license to display the preprint in perpetuity. It is made available under aCC-BY 4.0 International license.

Table S8.1 - Initial parameters (Cell Growth and Mechanical Constraints)

Parameter	Symbol	Simulation Value	Literature Ref.	Description
**Stem Cells**				
InitSTEM_LambdaSurface	$\lambda_{0s,stem}$	2.0	–	*How strongly stem cell regulates its surface area towards a desired size*
InitSTEM_TargetSurface	$S_{0target,stem}$	18.0	(49)	*The ideal surface area each stem cell tries to maintain. Defined to constraint cell shape*
InitSTEM_LambdaVolume	$\lambda_{0v,stem}$	2.0	–	*How strongly a stem cell regulates its volume towards a desired size.*
InitSTEM_TargetVolume	$V_{0target,stem}$	25.0	(83)	*The ideal volume each stem cell tries to maintain. Reference 10 -30 µm*
DensitySTEM_HalfMaxValue	$k_{mdensity,stem}$	125.0	*Fitted (S1.5)*	*Cell density of stem cells half-max growth response*
EGF_STEM_HalfMaxValue	$k_{mEGF,stem}$	3.5	*Fitted (S1.5)*	*EGF concentration stem cells achieve half-max growth response*
InitSTEM_LambdaChemo	$\lambda_{chemoMbias,stem}$	100.0	–	*How strongly stem cells move toward areas of higher chemoattract.*
**Basal Cells**				
InitBASAL_LambdaSurface	$\lambda_{0s,basal}$	2.0	–	*How strongly basal cell regulate its surface area towards a desired size*
InitBASAL_TargetSurface	$S_{0target,basal}$	20.0	(49)	*The ideal surface area each basal cell tries to maintain. Defined to constraint cell shape*
InitBASAL_LambdaVolume	$\lambda_{0v,basal}$	2.0	–	*How strongly a basal cell regulates its volume towards a desired size*
InitBASAL_TargetVolume	$V_{0target,basal}$	25.0	(83)	*The ideal volume each basal cell tries to maintain. Reference 10 -30 µm*
DensityBASAL_HalfMaxValue	$k_{mdensity,basal}$	125.0	*Fitted (S1.5)*	*Cell density of basal cells half-max growth response*
EGF_BASAL_HalfMaxValue	$k_{mEGF,basal}$	7.0	*Fitted (S1.5)*	*EGF concentration basal cells achieve half-max growth response*
InitBASAL_LambdaChemo	$\lambda_{chemoMbias,basal}$	1000.0	–	*How strongly basal cells move to areas with more chemoattractant*
**Wing Cells**				
InitWING_LambdaSurface	$\lambda_{0s,wing}$	5.0	–	*How strongly wing cell regulates its surface area towards a desired size*
InitWING_TargetSurface	$S_{0target,wing}$	25.0	(49)	*The ideal surface area each wing cell tries to maintain. Defined to constraint cell shape*
InitWING_LambdaVolume	$\lambda_{0v,wing}$	2.0	–	*How strongly a wing cell regulates its volume towards a desired size*
InitWING_TargetVolume	$V_{0target,wing}$	25.0	(83)	*The ideal volume each wing cell tries to maintain. Reference 10 -30 µm*
InitWING_EGFLambdaChemo	$\lambda_{chemoEGF,wing}$	20.0	(102)	*How strongly wing cells move toward regions with higher EGF*
**Superficial Cells**				
InitSUPER_LambdaSurface	$\lambda_{0s,super}$	5.0	–	*How strongly superficial cells regulate surface area to desired size*
InitSUPER_TargetSurface	$S_{0target,super}$	25.0	(49)	*The ideal surface area each superficial cell tries to maintain. Defined to constraint cell shape*
InitSUPER_LambdaVolume	$\lambda_{0v,super}$	5.0	–	*How strongly superficial cells regulate its volume to desired size*
InitSUPER_TargetVolume	$V_{0target,super}$	25.0	(83)	*Ideal volume superficial cell tries to maintain. Reference 10 -30 µm*

bioRxiv preprint doi: https://doi.org/10.1101/2025.08.11.669602; this version posted August 14, 2025. The copyright holder for this preprint (which was not certified by peer review) is the author/funder, who has granted bioRxiv a license to display the preprint in perpetuity. It is made available under aCC-BY 4.0 International license.

Table S8.2 - Initial parameters (Chemical Fields [EGF, SLS, Movement Bias])

Parameter	Symbol	Simulation Value	Literature Ref.	Description
**SLS Diffusion**				
SLS_STEMDiffCoef	$D_{chemstem}$	5.0	—	*How quickly the chemical spreads inside stem cells*
SLS_BASALDiffCoef	$D_{chembasal}$	5.0	—	*How quickly the chemical spreads inside basal cells*
SLS_WINGDiffCoef	$D_{chemwing}$	5.0	—	*How quickly the chemical spreads inside wing cells*
SLS_SUPERDiffCoef	$D_{chemsuper}$	5.0	—	*How quickly the chemical spreads inside superficial cells*
SLS_MEMBDiffCoef	$D_{chemmemb}$	5.0	—	*How quickly the chemical spreads in the Bowman's membrane (periphery)*
SLS_LIMBDiffCoef	$D_{chemlimb}$	5.0	—	*How quickly chemical spreads in the Bowman's membrane (limbal region)*
SLS_TEARDiffCoef	$D_{chemtear}$	5.0	—	*How quickly the chemical spreads in the tear layer*
SLS Global Decay	k_{dchem}	0.0	—	*For the model the only breakdown and inactivation of the chemical occurs by cell death where the amount inside the cell gets inactive as cells die and washed by the tear*
**EGF Diffusion / Uptake**				
EGF_SUPERDiffCoef	$D_{EGFsuper}$	20.0	(50,55)	*How quickly EGF diffuses (spreads) through superficial cells. The references show a limitation of space for EGF to defuse through the sides of the cells and it was shown that EGF applied in intact corneas do not proliferate as wounded ones*
EGF_FieldUptakeBASAL	$\mu_{EGFbasal}$	0.0	—	*How quickly basal cells consume EGF from their surroundings*
EGF_FieldUptakeSTEM	$\mu_{EGFstem}$	0.0	—	*How quickly stem cells consume EGF from their surroundings*
EGF_FieldUptakeSuper	$\mu_{EGFsuper}$	0.0	—	*How quickly superficial cells consume EGF*
EGF_FieldUptakeWing	$\mu_{EGFwing}$	0.0	—	*How quickly wing cells consume EGF*
EGF_ScreteAmount	$\varphi_{EGFtear}$	1.0	(35,54)	*The constant amount of EGF introduced into the system from tears. The value for secretion was normalized to have the max value of 1 throughout the simulated space.*
EGF_GlobalDecay	k_{dEGF}	0.5	(88)	*The overall rate at which EGF naturally breaks down (decays) over time. The values of EGF half-life from the reference was 78 min +- 36 min transformed into ≈ 0.1 per MCS, value used is higher to account for other things like uptake, evaporation and sequestration.*
**Movement Bias Field**				
MovementBiasScreteAmount	$\varphi_{Mbiasmemb, limb}$	1.0	—	*The constant amount of chemoattractant secreted by the boundary (Bowman's membrane) to guide cell movement.*
MovementBiasUptake	$\mu_{Mbiasbasal,stem}$	1.0	—	*How quickly basal cells absorb (take up) this chemoattractant*

bioRxiv preprint doi: https://doi.org/10.1101/2025.08.11.669602; this version posted August 14, 2025. The copyright holder for this preprint (which was not certified by peer review) is the author/funder, who has granted bioRxiv a license to display the preprint in perpetuity. It is made available under aCC-BY 4.0 International license.

Table S8.3 - Initial parameters (Injury Setup and Focal Point Plasticity Links)

Parameter	Symbol	Simulation Value	Literature Ref.	Description
SimTime	t_{final}	*50,400.0*	–	*How long the simulation runs before it stops (in MCS)*
**Injury Setup**				
InjuryTime	t_{injury}	*7200.0*	–	*The simulation time at which the injury event occurs*
SLS_X_Center	$x_{centerchem}$	*100*	–	*The x-coordinate of the center of the chemical source*
SLS_Y_Center	$y_{centerchem}$	*75*	–	*The y-coordinate of the center of the chemical source*
SLS_Concentration	c_{chem}	*[750 a.u.(slight), 1500 a.u.(mild), 2500 a.u.(mod.)]*	(3)	*The initial strength or concentration of the chemical introduced. This parameter was chosen based on the descriptions of different classifications following the depth of injury model*
InjuryX_Center	$x_{centerabla}$	*150*	–	*The x-coordinate of the ablation injury center*
InjuryY_Center	$y_{centerabla}$	*60*	–	*The y-coordinate of the ablation injury center*
InjuryRadius	π_{abla}	*25*	–	*How far from the center cells are affected by the ablation (a radius)*
**FPP Link Setup**				
LINKWALL_lambda_distance	$\lambda_{0_{link,wall}}$	*50*	–	*How strongly the link between superficial cells and simulation boundary maintains a set distance*
LINKWALL_target_distance	$L_{0_{targetlink,wall}}$	*3*	–	*Desired length of the link between superficial cells and the boundary*
LINKWALL_max_distance	$L_{m_{link,wall}}$	*1000*	–	*Max length link between superficial cells vs boundary can stretch before it breaks*
LINKSUPER_lambda_distance	$\lambda_{0_{link,super}}$	*50*	–	*How strongly the link between two superficial cells maintains a set distance*
LINKSUPER_target_distance	$L_{0_{target_{link,super}}}$	*3*	–	*The desired resting length of the link between superficial cells*
LINKSUPER_max_distance	$L_{m_{link,super}}$	*1000*	–	*Max length the link between superficial cells can stretch before it breaks*

bioRxiv preprint doi: https://doi.org/10.1101/2025.08.11.669602; this version posted August 14, 2025. The copyright holder for this preprint (which was not certified by peer review) is the author/funder, who has granted bioRxiv a license to display the preprint in perpetuity. It is made available under aCC-BY 4.0 International license.

Table S8.4 - Initial parameters (Simulation/Logging Controls)

Parameter	Symbol	Simulation Value	Description
GrowthControl	—	*True*	*Enable or disable cell growth processes for debugging*
MitosisControl	—	*True*	*Enable or disable cell division (mitosis) for debugging*
DeathControl	—	*True*	*Enable or disable cell death mechanisms for debugging*
DifferentiationControl	—	*True*	*Enable or disable cell differentiation processes for debugging*
CellCount	—	*True*	*Collect the number of cells over time*
PressureTracker	—	*False*	*Show a real-time plot of pressure in the simulation*
EGF_SeenByCell	—	*True*	*Track the EGF concentration experienced by each cell inside CC3D*
SLS_SeenByCell	—	*False*	*Track the chemical concentration experienced by each cell inside CC3D*
ThicknessPlot	—	*True*	*Collect and plot data on the tissue thickness over time*
SurfactantTracking	—	*False*	*Track chemical distribution over time*
SnapShot	—	*True*	*Take snapshots of the simulation state at regular intervals*
InjuryType	—	*True*	*Choose the type of injury (e.g., ablation or chemical)*
IsInjury	—	*False*	*Enable or disable the injury feature in the simulation*
SLS_Threshold	ω_{chem}	*True*	*The chemical concentration limit above which cells are considered dead*
SLS_Injury	—	*False*	*Enable or disable a chemical-based injury using SLS (for testing how chemicals spread and affect cells)*
SLS_Threshold_Method	—	*True*	*If enabled, cells will die when chemical level surpasses a certain threshold*
SLS_Gaussian_pulse	—	*True*	*Whether the chemical is introduced as a concentrated 'droplet' (Gaussian) or as a uniform 'coating'*
AutoAdjustLinks	—	*True*	*If enabled, the link properties are auto adjusted to keep the tension constant*
